# Cardiac autonomic function in bronchiectasis and age and gender-matched healthy participants: case–control study

**DOI:** 10.1038/s41598-026-36722-9

**Published:** 2026-02-03

**Authors:** Dhiya Dinesh, K. Vaishali, Anup Bhat, Mukesh Kumar Sinha, Amithash M. Prabhudev

**Affiliations:** 1 Department of Physiotherapy, Manipal College of Health Professions, Manipal Academy of Higher Education, Manipal, Karnataka 576104 India; 2 Department of Respiratory Medicine, Kasturba Medical College, Manipal Academy of Higher Education, Manipal, Karnataka 576104 India; 3 Present Address: Royal Papworth Hospital NHS Foundation Trust, Cambridge Biomedical Campus, Cambridge, UK; 4 Present Address: NMC Speciality Hospital, Abu Dhabi, United Arab Emirates

**Keywords:** Autonomic modulation, Autonomic nervous system, Chronic respiratory diseases, Heart rate variability, Sympathetic nervous system, Physiology, Biomarkers, Cardiology, Diseases, Risk factors

## Abstract

This study aims to evaluate and compare cardiac autonomic function, specifically through heart rate variability (HRV), between individuals with bronchiectasis and their age- and gender-matched healthy counterparts. This study employed a case-control design, involving 60 participants diagnosed with bronchiectasis (cases) and a control group of healthy individuals matched by age and gender. HRV data was collected over a five-minute interval, focusing on frequency domain parameters including total power, very low frequency (VLF), low frequency (LF), high frequency (HF), and the LF/HF ratio. The bronchiectasis group exhibited a significantly elevated LF/HF ratio, indicating a shift in cardiac sympathovagal balance, relative to the control group (2.25 ± 0.39 vs. 2.05 ± 0.38; *p* = 0.006). Additionally, marked differences were found in specific frequency domain parameters: LF (2.33 ± 0.55 vs. 2.55 ± 0.46; *p* = 0.021) and HF (2.06 ± 0.75 vs. 2.5 ± 0.56; *p* = 0.001). The results suggest a notable disturbance in cardiac autonomic regulation among individuals with bronchiectasis, compared to healthy individuals.

## Introduction

Bronchiectasis is a chronic and progressive lung disease characterized by permanent and abnormal dilatation of bronchial airways, which is often associated with chronic productive cough, purulent sputum, recurrent infection, airway inflammation, obstruction, and damage. ^1–3^ The prevalence rate of bronchiectasis in the general population is 39.9 cases per 100,000^4^ The prevalence increases with age and is considered to be an underestimate of the true burden of the disease^5^ It affects extremes of age, and it has a slight female preponderance^6^

Bronchiectasis, characterized by chronic airway inflammation and structural lung damage, is increasingly recognized as having systemic consequences beyond the respiratory system. The persistent inflammation associated with bronchiectasis can trigger a cascade of systemic effects, including the release of inflammatory mediators into the circulation^7–10^ These mediators can directly influence autonomic nervous system activity, leading to alterations in cardiac sympathovagal balance. Furthermore, the increased work of breathing and potential for intermittent hypoxemia may contribute to autonomic dysfunction through chemoreceptor activation and altered baroreceptor sensitivity^11^ However, the precise mechanisms linking bronchiectasis to cardiac autonomic dysfunction remain incompletely understood, warranting further investigation. Airflow limitation, systemic inflammation, hypoxemia, arterial stiffness, loss of elastic recoil, and use of bronchodilator drugs can trigger a series of cardiovascular adjustments. This, in turn, can affect the cardiac autonomic function in subjects with bronchiectasis^12^.

Cardiac autonomic dysfunction, a common disorder in bronchiectasis, especially in advanced stages, is a predictor of cardiovascular events and mortality, reflected by delayed heart rate recovery (HRR). It relates to reduced exercise capacity, frequent exacerbations, and increased cardiovascular risk.13 Bronchiectasis-Chronic Obstructive Pulmonary Disease Overlap Syndrome (BCOS) combines symptoms like cough and reduced tolerance, with treatments such as beta-agonists and muscarinic antagonists affecting heart rate and risk.14 Lung hyperinflation worsens cardiac function by increasing pressures, causing ventilation/perfusion mismatches and hypoxemia. Frequent exacerbations are linked to elevated C-reactive protein, arterial stiffness, and troponin levels, with the bronchiectasis severity index strongly correlating with cardiac risk markers, regardless of age^13,14^.

Cardiac autonomic function can be assessed with various measures like heart rate recovery, baroreflex sensitivity, heart rate variability (HRV), and catecholamine concentration in the blood^15^ HRV is a non-invasive, simple, reliable reflection of physiological factors modulating the normal rhythm of the heart and assessing the activities of the autonomic nervous system^16^ High HRV is a signal of efficient autonomic function seen in healthy individuals. People with high HRV may also have greater cardiorespiratory fitness and may be more resilient to stress; whereas a lower HRV indicates abnormal and insufficient adaptation of the cardiac autonomic function^17^.

Alongside the ventilatory limitations caused by the deterioration of lung structure, numerous disease-related factors (such as physical inactivity, hypoxemia, hypercapnia, exercise limitations, muscle impairment, and cachexia) may influence HRV. Moreover, individuals with respiratory dysfunction often experience heightened airway resistance, and this increased resistance can amplify the effort required for breathing, potentially affecting autonomic function^18^.

While the potential for autonomic dysfunction in bronchiectasis has been hypothesized, the specific impact on cardiac autonomic function, as measured by HRV, remains poorly defined. Therefore, this study aimed to evaluate and compare HRV parameters in individuals with bronchiectasis and healthy controls, with the goal of informing future strategies for managing cardiovascular health in this patient population.

## Methods

The study was approved by Kasturba Medical College and Kasturba Hospital Institutional Ethics Committee (IEC: 72/2018) and was registered under the Clinical Trial Registry of India (CTRI/2018/11/01,635). All procedures were conducted in accordance with applicable guidelines and regulations. Informed consent was obtained from all participants, who signed and returned the consent forms prior to their involvement in the study. The design and reporting of this observational study adhered to the Strengthening the Reporting of Observational Studies in Epidemiology guidelines for cross-sectional studies. (STROBE)^19^ Based on a power analysis a total sample size of 128 participants (64 per group) was determined. However, the study was completed with a total of 120 participants, which was sufficient to achieve the desired statistical power for the HRV variable of the LF/HF ratio, and the study was concluded accordingly. Participants with bronchiectasis were recruited from a tertiary care hospital (Kasturba Hospital, Manipal, Karnataka, India) between 4^th^ December 2018 to March 2019. The inclusion criteria specified male and female subjects aged 18–80 years with a confirmed diagnosis of bronchiectasis via high-resolution computed tomography (CT) scans.

Eligible individuals were required to have a body mass index (BMI) within the range of 18–25 kg/m^2^, be clinically stable with a modified Medical Research Council (mMRC) breathlessness score of grade I or II, and not have experienced a recent hospital admission in the prior week. This criterion was implemented to ensure that participants were in a clinically stable phase, minimizing the potential influence of acute illness or recent medical interventions on HRV parameters. While a longer hospitalization-free period (e.g., 4 weeks) might provide a more stringent definition of stability, we chose a 1-week period to balance the need for clinical stability with the feasibility of participant recruitment. We anticipated that a longer period would significantly reduce the number of eligible participants. This BMI range was selected to exclude individuals with significant underweight (BMI < 18.0 kg/m^2^) or obesity (BMI > 25.0 kg/m^2^), as both of these conditions can independently influence HRV^16^ . Exclusion criteria included the presence of cardiac conditions including coronary artery disease, heart failure, and a history of lung surgeries. Additional exclusions were individuals with diagnosis of cancer, hypertension, pulmonary artery hypertension (PAH), or Type II diabetes mellitus. The healthy control group was recruited through word of mouth, caregiver referrals, and community contacts, and those with a known history of any systemic illness were excluded from the control group. Prior to their participation, all participants signed the written informed consent.

Before initiating the procedure, we collected a comprehensive set of demographic and clinical information from each participant. We measured height and weight to calculate body mass index (BMI). Additionally, we recorded heart rate, systolic, and diastolic blood pressure to establish baseline vitals. Peripheral capillary oxygen saturation (SpO2) levels were monitored to determine the oxygen saturation of peripheral blood. Furthermore, we gathered detailed accounts of each participant’s drug history, including all medications taken and smoking history, to account for any confounding factors that might affect the study outcomes.

### HRV measurement

Cardiac autonomic function was evaluated using the Polar RS 800 CX radio telemetric heart rate monitor, which measures standard time and frequency domain parameters of HRV. Assessment of HRV was carried out in accordance with the Task recommendation^20^ . The assessment occurred under controlled conditions, with a temperature range of 21–24 °C and humidity maintained between 50 and 60%. Participants were asked to refrain from consuming caffeine or alcohol for at least 12 h prior to the assessment. They were also instructed to avoid heavy meals within 2 h of the measurement. The timing of medication use relative to the HRV measurement was not recorded. Additionally, their last bronchodilator dose was not recorded prior to the HRV assessment; however, all participants’ recordings were completed before 10 AM.

Before commencing the measurement, participants received a detailed explanation of the procedures and were instructed to relax, breathe normally, avoid movements and conversation during the test. Following the initial evaluation, heart monitor straps were securely placed on the participants’ chests, positioned over the distal third of the sternum. Participants were then asked to lie in a supine position for a 15-min rest period. After this resting phase, the heart rate receiver (radio telemetric heart rate monitor) was positioned on the participants’ wrists, and HRV data were recorded for a duration of five minutes. The frequency domain measures of HRV analyzed included total power, very low frequency (VLF), low frequency (LF), high frequency (HF), and the LF/HF ratio^20–22^ . Data was collected between 8 a.m. and 11 a.m. to minimize the impact of circadian rhythms on the measurements. HRV data were analyzed using Polar ProTrainer software. This software package was utilized to derive both time and frequency domain parameters of heart rate variability from the recordings obtained via the Polar RS 800 CX radio telemetric heart rate monitor. The software incorporated validated algorithms for the accurate calculation of key HRV metrics, including total power, very low frequency (VLF), low frequency (LF), high frequency (HF), and the LF/HF ratio, thus ensuring the reliability and reproducibility of the analysis.

### Statistical analysis

SPSS version 15.0 (released in 2006; SPSS for Windows, SPSS Inc., Chicago, IL, USA) was utilized for data compilation and analysis. Descriptive statistics were employed to characterize the demographic details of the participants. To evaluate the normality of the data, the Shapiro–Wilk test was performed. An independent t-test was used to compare the HRV indices between the bronchiectasis group and healthy participants. Due to the skewness in the data, a log transformation was applied. Based on a power analysis conducted using GPower 3.1, a total sample size of 128 participants (64 per group) was determined to be necessary to detect a moderate effect size (d = 0.5) with 80% power at a 0.05 significance level. Differences between the two groups were considered statistically significant when the p value was ≤ 0.05.

## Results

**Fig. 1:**
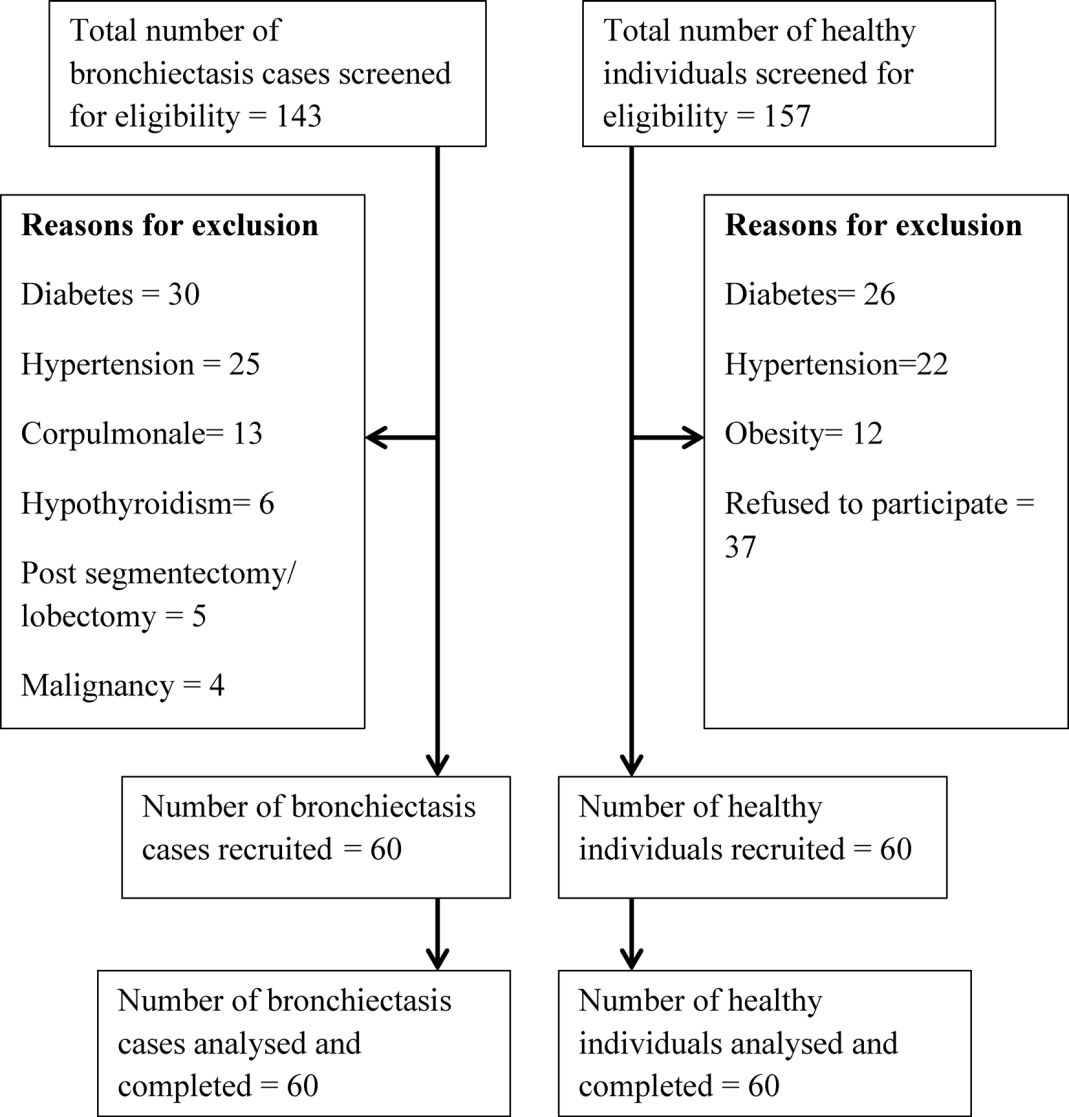
Participant recruitment process.

In this study, we analyzed HRV parameters in individuals with bronchiectasis compared to healthy control subjects. A total of 300 participants were screened for eligibility, comprising 143 individuals with bronchiectasis and 157 healthy participants. (Fig. 1) Post screening, a total of sixty participants in each group were recruited into the study.

Table 1 presents the comparable baseline demographics for both the bronchiectasis and healthy groups. Notably, significant differences were identified in heart rate, diastolic blood pressure, SpO_2_ levels, and BMI between the two groups. The bronchiectasis group (n = 60) had a mean age of 51.10 years, while the healthy control group (n = 60) had a mean age of 48.98 years; this difference was not statistically significant (*p* = 0.34). The gender distribution was similar in both groups, with 26 (43%) males and 34 (57%) females. However, significant differences were observed in heart rate (83.30 bpm in bronchiectasis vs. 70.36 bpm in healthy, *p* = 0.01), diastolic blood pressure (79.96 mmHg vs. 10.44 mmHg, *p* = 0.012), SpO2 (96.86% vs. 98.71%, *p* = 0.01), and Body Mass Index (20.20 vs. 22.64, *p* = 0.01).

**Table 1:** Baseline characteristics of subjects with bronchiectasis and healthy participants.

Variables	Mean ± standard deviation		*p* value
	Bronchiectasis cases n = 60	Healthy participants n = 60	
Age in years	51.10 ± 12.73	48.98 ± 11.64	0.34
Gender (male: female)N(%)	26(43):34(57)	26(43):34(57)	
Heart rate	83.30 ± 12.79	70.36 ± 9.89	0.01
Systolic blood pressure	117.30 ± 17.33	115.26 ± 9.90	0.43
Diastolic blood pressure	79.96 ± 8.79	10.44 ± 8.79	.012
SpO_2_	96.86 ± 2.01	98.71 ± 1.10	0.01
Body mass index	20.20 ± 3.02	22.64 ± 2.66	0.01
Smoking history (Nonsmokers: smokers) N (%)	54(90):6(10)	57(95):3(5)	

Table 2 details the characteristics of the bronchiectasis group (n = 60). Regarding disease severity, the majority (60%) had mild bronchiectasis, with 28% classified as moderate and 12% as severe. A significant proportion (45%) reported a positive history of exposure to biomass smoke. In terms of medication use, 65% were using long-acting beta-agonists, 25% short-acting beta-agonists, and 25% corticosteroids.

**Table 2:** Characteristics of individuals with bronchiectasis.

Severity of bronchiectasis (FACED)	N(%)
Mild	37(60)
Moderate	16(28)
Severe	7(12)

Drug history	Number N (%)
Long-acting beta-agonists	39(65)
Short-acting beta agonists	15(25)
Corticosteroids	15(25)
ATT drugs	1(2)
Xanthine derivative bronchodilators	10(18)
Expectorants	26(43)
Antitussives	26(43)
Antihistamines	31(58)
Antibiotics	25(42)
Mucolytics	4(7)
Years since diagnosis	
Less than 1 year	17(28)
1–5 years	23(39)
More than 5 years	20(33)
Positive history of exposure to biomass smoke	27(45)

Table 3 presents a comparison of frequency domain heart rate variability (HRV) parameters between individuals with bronchiectasis and healthy participants. The data, log-transformed, shows that the bronchiectasis group had lower values for all frequency domain measures: total power, VLF (very low frequency), LF (low frequency), and HF (high frequency). Notably, the differences in total power (*p* = 0.037), LF (*p* = 0.021), and HF (*p* = 0.006) reached statistical significance, indicating reduced overall HRV and potentially altered autonomic nervous system function in the bronchiectasis group. (Table 3).

**Table 3:** Comparison of frequency domain parameters between individuals with bronchiectasis and healthy participants (log transformed data).

Frequency domain variables	Individuals with bronchiectasis (n = 60)	Healthy participants (n = 60)	*p*-value
Total power	3.07 ± 0.53	3.26 ± 0.44	0.037*
VLF	2.88 ± 0.52	3.0 ± 0.46	0.201
LF	2.33 ± 0.55	2.55 ± 0.46	0.021*
HF	2.06 ± 0.75	2.5 ± 0.56	0.001*
LF/HF ratio	2.25 ± 0.39	2.05 ± 0.38	0.006*

VLF, very low frequency; LF, low frequency; HF, high frequency and low frequency/high frequency ratio. *Significant difference between individuals with bronchiectasis and healthy participants *p* ≤ 0.05.

This study also compares HRV metrics, specifically standard deviation of the NN intervals (SDNN) and root mean square of successive differences between normal heartbeats (RMSSD), between individuals with bronchiectasis and healthy participants. The SDNN, which measures the overall variability in heart rate, was slightly lower in individuals with bronchiectasis (mean of 1.55 ms) compared to healthy participants (mean of 1.63 ms). However, the p-value of 0.09 indicates that this difference is not statistically significant, suggesting that the overall HRV does not differ substantially between the two groups. Similarly, the RMSSD, a metric for short-term HRV influenced by the parasympathetic nervous system, was also lower in individuals with bronchiectasis (mean of 1.36 ms) compared to healthy participants (mean of 1.50 ms). The *p*-value of 0.13 suggests that this difference is also not statistically significant. Therefore, the study concludes that there are no significant differences in both overall and short-term HRV between individuals with bronchiectasis and healthy controls, as the observed variations do not reach statistical significance at the conventional threshold of 0.05. (depicted in Table 4).

**Table 4:** Comparison of time domain parameters between individuals with bronchiectasis and healthy participants (log transformed data).

Frequency domain variables	Individuals with bronchiectasis (n = 60)	Healthy participants (n = 60)	*p*-value
SDNN	1.55 ± 0.30	1.63 ± 0.22	0.09
RMSSD	1.36 ± 0.52	1.50 ± 0.31	0.13

SDNN: standard deviation of the NN intervals.

RMSSD: root mean square of successive differences between normal heartbeats.

## Discussion

The findings of the present study indicate a notable derangement in HRV parameters among individuals with bronchiectasis compared to age-matched healthy participants. This study aimed to elucidate the differences in cardiac autonomic function between these two groups, revealing significant changes in the frequency domain parameters of HRV in the bronchiectasis population. Such alterations suggest a sympathovagal imbalance that could have important clinical implications.

Specifically, our results demonstrated a decrease in HF power of HRV in individuals with bronchiectasis, suggesting a reduction in parasympathetic activity. This observation aligns with prior research conducted on individuals with COPD and obstructive sleep apnea, where decreased HF was attributed to factors such as higher respiratory rates and lung hyperinflation^23^ . However, it is noteworthy that participants in our bronchiectasis group did not exhibit an elevated respiratory rate, indicating that other mechanisms may be contributing to the observed reductions in parasympathetic activity. The lack of an assessment for hyperinflation in our bronchiectasis group may be considered as a limitation of the current study. The etiologies of bronchiectasis among participants were primarily identified as post-infectious sequelae for a predominant proportion of cases. Smaller subsets were attributed to Allergic Bronchopulmonary Aspergillosis (ABPA) or were idiopathic in origin. While Post-tuberculosis (TB) lung disease is recognized for its potential cardiovascular implications^24–26^ , the specific relationship between post-TB lung disease and Heart Rate Variability (HRV) remains underexplored. Although active TB, especially in advanced stages, may induce systemic inflammation, immune activation, and atherogenesis, thereby elevating the risk of cardiac complications^24,25,27^, empirical evidence directly linking post-TB lung disease to alterations in HRV is currently limited in the published literature.

Moreover, significant differences were found in the LF and LF/HF powers of HRV. The LF power reflects sympathetic afferent activity, while the LF/HF ratio serves as a measure of sympathovagal balance. Preliminary findings by Rached et al. support our results, highlighting similar patterns in individuals with bronchiectasis^12^ . This alteration in sympathetic activity can be attributed to hypercapnia, recurrent hypoxemia resulting in increased airway obstruction, increased respiratory effort, use of beta sympathomimetics and systemic inflammation , as explained among individuals with COPD^28^ . Although our participants with bronchiectasis were not hypoxemic, they did present with relatively reduced oxygen saturation levels compared to healthy controls, suggesting that even subtle variations in oxygenation could influence autonomic regulation^28^ . Also, in our study, participants could have concomitant respiratory conditions, such as chronic obstructive pulmonary disease (COPD), bronchial asthma (BA), or other obstructive airway diseases (OAD). The decision for inclusion was based on the understanding that bronchiectasis frequently coexists with other respiratory disorders in clinical practice, reflecting a 'real-world’ patient population. While the presence of comorbidities may introduce heterogeneity, excluding these patients would limit the generalizability of our findings to the broader population of individuals with bronchiectasis. We acknowledge that the inclusion of patients with COPD, asthma, or other airway diseases may confound the interpretation of HRV parameters. To mitigate this potential bias, we collected all the details of comorbidities.

Given the established influence of stress, anxiety, depression, and other psychiatric disorders on HRV parameters ^29–32^, it is possible that the observed differences in HRV between the bronchiectasis and control groups were, in part, influenced by undiagnosed or unquantified mental health factors. While we did not specifically exclude participants based on mental health status, future research should consider incorporating validated screening tools for anxiety, depression, and other relevant mental health conditions to better control for these potential confounders. Additionally, the differences in BMI observed between bronchiectasis patients and healthy controls may be affected by the selection criteria used for both groups. However, we established specific BMI thresholds for inclusion in our study. Future research could improve comparability by applying consistent inclusion and exclusion criteria across both groups, which would help reduce potential discrepancies and more accurately assess the impact of bronchiectasis on BMI.

In addition, the bronchiectasis group exhibited a higher baseline heart rate and reduced HRV, reflecting altered cardiac autonomic modulation characterized by increased sympathetic tone at rest. This alteration in sympathovagal balance may be related to chronic respiratory failure, sustained intermittent hypoxia, oxidative stress, and lung hyperinflation^33^, along with arousal from sleep, all of which contribute to the loss of respiratory sinus arrhythmia^28^. These factors are recognized associations with heightened sympathetic activity, further underscoring the impact of bronchiectasis on cardiac autonomic regulation. Additionally, while our findings aligned with the autonomic changes observed in COPD and OSA, the absence of hyperinflation or elevated respiratory rates in our bronchiectasis group suggested distinct mechanisms. We propose that the chronic inflammation inherent to bronchiectasis significantly contributed to this autonomic imbalance, mirroring links between inflammatory markers and HRV in other respiratory diseases^34^. Additionally, ongoing airway remodeling^35^, oxidative stress, and altered reflex control^36^ likely played roles in shifting sympathovagal balance, even without overt hyperinflation.

### Clinical significance

The observed reduction in HRV in individuals with bronchiectasis carries potential clinical implications. Reduced HRV is often associated with increased cardiovascular risk and mortality in various populations. These findings suggest that evaluating HRV in bronchiectasis patients as a potential marker of autonomic dysfunction and increased cardiovascular risk. Monitoring HRV could aid in identifying patients who may benefit from interventions aimed at improving autonomic balance, such as pulmonary rehabilitation programs incorporating exercise and breathing techniques, or pharmacological strategies targeting underlying inflammation. Furthermore, these HRV parameters may serve as a non-invasive tool for assessing disease severity and predicting prognosis in bronchiectasis, potentially guiding more personalized and proactive management strategies.

However, this study was not without limitations. A significant constraint was the inability to control or restrict bronchodilator use among participants, which could have influenced HRV outcomes. Furthermore, the inclusion of individuals with concomitant respiratory diseases introduced heterogeneity and necessitated caution when generalizing findings. Other limitations included the lack of disease severity stratification and objective lung function testing in controls. Future research should aim to address these by involving larger, more diverse, and homogeneous populations, employing longitudinal study designs, and incorporating controlled medication protocols. Such studies could further refine the potential of autonomic dysfunction as a therapeutic target and investigate the relationship between HRV, functional capacity, and physical activity levels in bronchiectasis. Another notable methodological limitation concerned our definition of clinical stability. While a pragmatic 1-week post-hospitalization criterion was adopted for recruitment feasibility, this diverged from the established expert consensus recommending a minimum 4-week period. Consequently, some participants, despite meeting our enrollment criteria, may have been in a recovery phase from a recent exacerbation, which could have subtly influenced their assessed heart rate variability parameters. Additionally, a methodological limitation was noted, as we did not record the precise timing of the last bronchodilator dose relative to the heart rate variability (HRV) assessment.

## Conclusion

This study shows altered cardiac autonomic function in individuals with bronchiectasis, compared to age- and gender-matched healthy participants. The findings reveal reduced parasympathetic activity, indicated by decreased HF power of HRV, along with increased sympathetic tone as evidenced by elevated LF power. Overall, these results underscore the need for further investigation into the mechanisms of autonomic dysregulation in bronchiectasis to inform the development of targeted management strategies addressing cardiovascular health in this population.

## Data Availability

Research data will be available from the corresponding author with a reasonable request.

## Abbreviations

HRV: Heart rate variability
COPD: Chronic obstructive pulmonary diseases
BA: Bronchial asthma
PAH: Pulmonary artery hypertension
OSA: Obstructive sleep apnea
SpO2: Saturation of peripheral oxygen
SDNN: Standard deviation of all NN intervals
RMSSD: The square root of the mean of the sum of the squares of differences between adjacent NN intervals
pNN50: Percentage of successive RR intervals that differ by more than 50 ms
VLF: Power in very low-frequency range
LF: Power in low frequency range
HF: Power in high frequency range
LF/HF: Ratio LF/HF

## References

[1] Barker, A. Medical progress: Bronchiectasis. *N. Engl. J. Med.* **346**, 1383–1393 (2002). 11986413 10.1056/NEJMra012519

[2] Chang, A. & Bilton, D. Exacerbations of cystic fibrosis: Non-cystic fibrosis bronchiectasis. *Thorax* **63**, 269–276 (2008). 18308962 10.1136/thx.2006.060913

[3] Khoo, J. K. et al. Bronchiectasis in the last five years: New developments. *J. Clin. Med.* **5**, 115 (2016). 27941638 10.3390/jcm5120115 PMC5184788

[4] Polverino, E. et.al. Global burden of non-cystic fibrosis bronchiectasis: A simple epidemiological analysis. *ERS* 1–2 (2012).

[5] EMBARC- The European Bronchiectasis Registry. What is Bronchiectasis? [Internet] [cited June 26, 2014] Available from: https://www.bronchiectasis.eu/what-is-bronchiectasis.

[6] McDonnell, M. J., Ward, C., Lordan, J. L. & Rutherford, R. M. Non cystic fibrosis bronchiectasis. *Q. J. Med.* **106**, 709–715 (2013). 10.1093/qjmed/hct109 23728208

[7] Cantin, A. M. Bronchiectasis: From targets to therapies. *Can. J. Respir., Critical Care, Sleep Med.* **7**(4), 206–212 (2023).

[8] King, P. T. The role of the immune response in the pathogenesis of bronchiectasis. *Biomed. Res. Int.* **2018**(1), 6802637 (2018). 29744361 10.1155/2018/6802637 PMC5878907

[9] Johnson, E., Long, M. B. & Chalmers, J. D. Biomarkers in bronchiectasis. *Eur. Respir. Rev.* **33**(173), 230234 (2024). 38960612 10.1183/16000617.0234-2023 PMC11220624

[10] Doumat, G., Aksamit, T. R. & Kanj, A. N. Bronchiectasis: A clinical review of inflammation. *Respir. Med.* **25**, 108179 (2025). 10.1016/j.rmed.2025.108179 40425105

[11] Giannoni, A. et al. Autonomic and respiratory consequences of altered chemoreflex function: clinical and therapeutic implications in cardiovascular diseases. *Eur. J. Heart Fail.* **25**(5), 642–656 (2023). 36907827 10.1002/ejhf.2819 PMC10989193

[12] Rached, S., Amaral, T. S., Angelis, K. D., Sartori, M. Abnormal heart rate variability in patients with bronchiectasis*. ERS* (2015).

[13] Sáez-Pérez, J. A. et al. Heart rate recovery after the 6-min walk test in people with bronchiectasis. *ERJ Open Res.* **11**(2), 00694 (2025). 40040890 10.1183/23120541.00694-2024 PMC11873979

[14] Alam, M. A. et al. Bronchiectasis-COPD overlap syndrome: A comprehensive review of its pathophysiology and potential cardiovascular implications. *Ther. Adv. Pulmon. Crit. Care Med.* **19**, 29768675241300810 (2024). 10.1177/29768675241300808 PMC11626662 39655338

[15] Achten, J. & Jeukendrup, A. E. Heart rate monitoring: Applications and limitations. *Sports Med.* **33**, 517–538 (2003). 12762827 10.2165/00007256-200333070-00004

[16] Acharya, U. R. et al. *Heart rate variability: a review: Med Biol Eng Comput.* **44**, 1031–1051 (2006). 17111118 10.1007/s11517-006-0119-0

[17] Vanderlei, L. C. et al. Basic notions of heart rate variability and its clinical applicability. *Rev. Bras. Cir. Cardiovasc.* **24**, 205–217 (2009). 19768301 10.1590/s0102-76382009000200018

[18] Camillo, C. A. et al. Heart rate variability and disease characteristics in patients with COPD. *Lung* **186**, 393–401 (2008). 18815834 10.1007/s00408-008-9105-7

[19] Von Elm, E. et al. The strengthening the reporting of observational studies in epidemiology (STROBE) statement: guIdelines for reporting observational studies. *The Lancet.* **370**(9596), 1453–1457 (2007). 10.1016/S0140-6736(07)61602-X 18064739

[20] Task Force of the European Society of Cardiology and the North American Society of Pacing and Electrophysiology. Heart rate variability: standards of measurement, physiological interpretation and clinical use. *Circulation* **93**,1043–1065 (1996). 8598068

[21] Porto, L. G. G. & Junqueira Júnior, L. F. Comparison of time-domain short-term heart interval variability analysis using a wrist-worn heart rate monitor and the conventional electrocardiogram. *PACE* **32**, 43–51 (2008). 10.1111/j.1540-8159.2009.02175.x 19140912

[22] Vanderlei, L. C., Silva, R. A., Pastre, C. M., Azevedo, F. M. & Godoy, M. F. Comparison of the Polar S810i monitor and the ECG for the analysis of heart rate variability in the time and frequency domains. *Braz. J. Med. Biol. Res.* **41**, 854–859 (2008). 18853042 10.1590/s0100-879x2008005000039

[23] Montemurro, L. T. et al. Cardiac sympathetic hyperactivity in patients with chronic obstructive pulmonary disease and obstructive sleep apnea. *COPD* **13**, 706–711 (2016). 27383268 10.1080/15412555.2016.1199668

[24] Marcu, D. T. et al. Cardiovascular involvement in tuberculosis: From pathophysiology to diagnosis and complications—a narrative review. *Diagnostics.* **13**(3), 432 (2023). 36766543 10.3390/diagnostics13030432 PMC9914020

[25] Katicheva, A. V. et al. Assesment of premarure death in patients with pulmonary tuberculosis and chronic obstructive pulmonary disease. *Bull. Russ. Military Med. Acad.* **22**(2), 19–22 (2020).

[26] Mitroi, D. M. et al. Hypercoagulability in tuberculosis: Pathophysiological mechanisms, associated risks, and advances in management—a narrative review. *J. Clin. Med.* **14**(3), 762 (2025). 39941433 10.3390/jcm14030762 PMC11818899

[27] George, P. J. et al. Coincident helminth infection modulates systemic inflammation and immune activation in active pulmonary tuberculosis. *PLoS Negl. Trop. Dis.* **8**(11), e3289 (2014). 25375117 10.1371/journal.pntd.0003289 PMC4222842

[28] Van Gestel, A. J. & Steier, J. Autonomic dysfunction in patients with chronic obstructive pulmonary disease (COPD). *J Thorac Dis.* **2**, 215–222 (2010). 22263050 10.3978/j.issn.2072-1439.2010.02.04.5 PMC3256465

[29] Liu, Z., Wu, H. & Hu, M. Heart rate variability in patients with generalized anxiety disorder and depressive disorder. *Chin. General Pract.* **22**(33), 4069–4072 (2019).

[30] Chen, L. F. et al. Depression, anxiety, and heart rate variability: a case-control study in Taiwan. *J. Med. Sci.* **34**(1), 9–18 (2014).

[31] Liu, W., Wang, S., Gu, H. & Li, R. Heart rate variability, a potential assessment tool for identifying anxiety, depression, and sleep disorders in elderly individuals. *Front. Psych.* **23**(16), 1485183 (2025). 10.3389/fpsyt.2025.1485183 PMC11798971 39916745

[32] Schiweck, C., Piette, D., Berckmans, D., Claes, S. & Vrieze, E. Heart rate and high frequency heart rate variability during stress as biomarker for clinical depression. A systematic review. *Psychol. Med.* **49**(2), 200–211 (2019). 30134999 10.1017/S0033291718001988

[33] Chen, W. L., Chen, G. Y. & Kuo, C. D. Hypoxemia and autonomic nervous dysfunction in patients with chronic obstructive pulmonary disease. *Respir. Med.* **100**(9), 1547–1553 (2006). 16488587 10.1016/j.rmed.2006.01.006

[34] Corbo, G. M. et al. C-reactive protein, lung hyperinflation and heart rate variability in chronic obstructive pulmonary disease–a pilot study. *COPD J. Chronic Obstr. Pulmon. Dis.* **10**(2), 200–207 (2013). 10.3109/15412555.2012.710667 22946790

[35] Leigh, R. et al. Human rhinovirus infection enhances airway epithelial cell production of growth factors involved in airway remodeling. *J. Allergy Clin. Immunol.* **121**(5), 1238–1245 (2008). 18355907 10.1016/j.jaci.2008.01.067

[36] Bernardi, L., Porta, C., Gabutti, A., Spicuzza, L. & Sleight, P. Modulatory effects of respiration. *Auton. Neurosci.* **90**(1–2), 47–56 (2001). 11485292 10.1016/S1566-0702(01)00267-3

